# Supplementary material for: Long-Term Outcomes of Breast Cancer Patients Receiving Levobupivacaine Wound Infiltration or Diclofenac for Postoperative Pain Relief
Source: Pharmaceutics. 2023 Aug 23;15(9):2183. doi: 10.3390/pharmaceutics15092183 (PMC10534840; doi:10.3390/pharmaceutics15092183)
Supplement: Supplementary file 1 [file pharmaceutics-15-02183-s001.zip › pharmaceutics-2492781-supplementary-revised.pdf]

pharmaceutics

MDPI

Article –

SUPPLEMENTAL FILE I for:

Long-term outcomes of breast cancer patients receiving levobupivacaine wound infiltration or diclofenac for postoperative pain relief

Josipa Glavas Tahtler[1,2], Dajana Djapic[1,2], Marina Neferanovic[2], Jelena Miletic[2], Marta Milosevic[2], Kristina Kralik[2], Nenad Neskovic[1,2*], Ilijan Tomas[2,3], Dora Mesaric[2,3], Ksenija Marjanovic[2,4]; Jasmina Rajc[2,4]; Zelimir Orkic[2,5], Ana Cicvaric[1,2] and Slavica Kvolik[1,2*]

[1]Department of Anesthesiology, resuscitation and Intensive Care, Osijek University Hospital; 31000 Osijek, Croatia
[2]Faculty of Medicine Osijek, Josip Juraj Strossmayer University of Osijek, 3100 Osijek, Croatia.
[3]Department of Oncology and Radiotherapy, Osijek University Hospital; 3100 Osijek, Croatia.
[4]Department of Pathology and Forensic Medicine, Osijek University Hospital, 1000 Osijek, Croatia
[5]Department of Surgery, Osijek University Hospital, 31000 Osijek, Croatia
*Correspondence: *SK skvolik@mefos.hr; *NN neskovic@mefos.hr

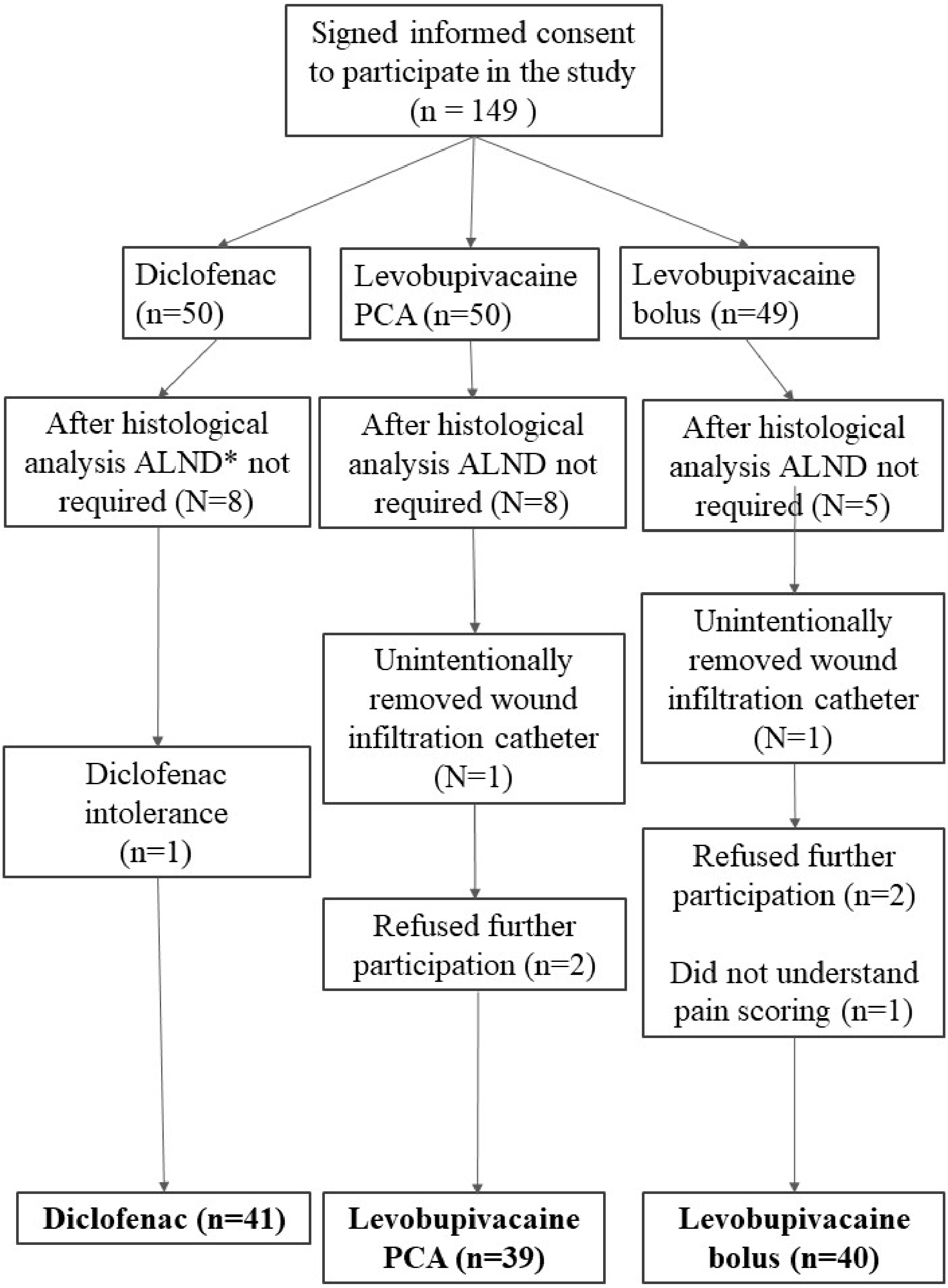

ALND* axillary lymph node dissection, PCA patient-controlled analgesia

Figure S1. Study flow chart for Effects of Postoperative Wound Infiltration Analgesia with Bupivacaine or Diclofenac on Long-Term Outcomes in Patients After Breast Cancer Surgery, randomized clinical trial

Table S1 – Postoperative pain score through 4 postoperative days in rest and movement measured with Numerical Rating Scale (NRS)

Postoperative day	Diclofenac (N=41)	$P^{\dagger}$	Levobupivacaine bolus (N=39)	$P^{\dagger}$	Levobupivacaine PCA (N=40)	$P^{\dagger}$	$P^{\ddagger}$
**NRS1-R**	1 (0 – 2)	**<0.001**	1 (0 – 2)	**<0.001**	0 (0 – 1)	**<0.001**	0.105
**NRS2-R**	0 (0 – 0.5)		0 (0 – 0)		0 (0 – 0)		0.138
**NRS3-R**	0 (0 – 0)		0 (0 – 0)		0 (0 – 0)		0.097
**NRS4-R**	0 (0 – 0)		0 (0 – 0)		0 (0 – 0)		0.791
**NRS1-M**	3 (1.5 – 4)	**<0.001**	3 (2 – 4)	**<0.001**	3 (2 – 3)	**<0.001**	0.783
**NRS2-M**	1 (1 – 3)		1 (0 – 2)		1 (1 – 2)		0.473
**NRS3-M**	1 (0.5 – 2)		1 (0 – 1)		0 (0 – 1.8)		**0.043**
**NRS4-M**	0 (0 – 1)		0 (0 – 1)		0 (0 – 1)		0.630

NRS in the postoperative days 1-4 was measured in rest (NRS 1-4R), and in the movement (NRS1-4M), values are presented as median (interquartile range); † Friedman's test; ‡ Kruskal Wallis's test.

Table S2 Significance of differences in domains of health status before and one year after surgery within groups based on Short Form 36 Health Survey (SF-36)

	**Domains of health status**	Diclofenac	Levobupivacaine bolus	Levobupivacaine PCA
		**Groups of patients**		
		P *	*P* *	*P* *
**Physical health**	Physical functioning	0.393	**0.003**	**0.008**
	Role limitation due to physical problems	0.180	0.082	0.692
	Bodily pain	0.087	**0.042**	0.325
	General health perception	0.508	0.159	0.365
**Mental health**	Energy - vitality	0.100	0.084	0.298
	Social functioning	0.923	0.096	**0.046**
	Role limitation due to emotional problems	0.731	0.568	0.673
	General mental health	0.657	0.721	0.793

*Wilcoxon test

Table S3. Shoulder pain examined with a shoulder disability questionnaire in patients with diclofenac analgesia before and one year after surgery.

	Before surgery N (%)			One-year follow-up N (%)			
	NA	No	Yes	NA	No	Yes	P^*
I wake up at night because of shoulder pain.	1 (2.5)	34 (85)	5 (12.5)	1 (4.8)	14 (66.7)	6 (28.6)	0.378
My shoulder hurts when I lie on it.	1 (2.4)	32 (78)	8 (19.5)	1 (4.8)	13 (61.9)	7 (33.3)	0.295
Because of pain in my shoulder, it is difficult to put on a coat or a sweater	0	36 (87.8)	5 (12.2)	0	19 (86.4)	3 (13.6)	0.260
My shoulder hurts during my usual daily activities	2 (4.9)	33 (80.5)	6 (14.6)	0	17 (77.3)	5 (22.7)	0.070
My shoulder hurts when I lean on my elbow or hand	2 (4.9)	38 (92.7)	1 (2.4)	1 (4.5)	17 (77.3)	4 (18.2)	0.156
My shoulder hurts when I move my arm.	2 (4.9)	39 (95.1)	0	1 (4.5)	20 (90.9)	1 (4.5)	**0.005**
My shoulder hurts when I write or type.	3 (7.3)	38 (92.7)	0	0	20 (95.2)	1 (4.8)	0.143
My shoulder is painful when I hold the driving wheel of my car or handle bars of my bike.	3 (7.5)	36 (90)	1 (2.5)	4 (20)	14 (70)	2 (10)	**<0.001**
When I lift and carry something my shoulder hurts.	2 (4.9)	30 (73.2)	9 (22)	0	13 (59.1)	9 (40.9)	**0.043**
During reaching and grasping above shoulder level my shoulder hurts.	2 (4.9)	33 (80.5)	6 (14.6)	0	15 (68.2)	7 (31.8)	**0.023**
My shoulder is painful when I open or close a door.	2 (4.9)	36 (87.8)	3 (7.3)	1 (4.5)	20 (90.9)	1 (4.5)	**<0.001**
My shoulder is painful when I bring my hand to the back of my head	2 (5)	33 (82.5)	5 (12.5)	0	16 (76.2)	5 (23.8)	0.568
My shoulder is painful when I bring my hand to my buttock	1 (2.4)	35 (85.4)	5 (12.2)	0	18 (81.8)	4 (18.2)	0.700
My shoulder is painful when I bring my hand to my low back.	2 (5)	34 (85)	4 (10)	0	16 (72.7)	6 (27.3)	0.706
I rub my painful shoulder more than once during the day.	0	38 (92.7)	3 (7.3)	0	17 (77.3)	5 (22.7)	0.227
Because of my shoulder pain I am more irritable and bad tempered with people than usual.	1 (2.5)	38 (95)	1 (2.5)	0	17 (77.3)	5 (22.7)	0.151

* marginal homogeneity test. Statistically significant differences in the shoulder pain during typical movements are bolded.

Table S4. Shoulder pain examined with a shoulder disability questionnaire in patients with levobupivacaine bolus analgesia before and one year after surgery.

	Before surgery N (%)			One-year follow-up N (%)			*P**
	NA	No	Yes	NA	No	Yes	
I wake up at night because of shoulder pain.	0	35 (92.1)	3 (7.9)	0	21 (80.8)	5 (19.2)	0.091
My shoulder hurts when I lie on it.	2 (5.3)	30 (78.9)	6 (15.8)	2 (8)	17 (68)	6 (24)	**0.011**
Because of pain in my shoulder, it is difficult to put on a coat or a sweater	0	37 (97.4)	1 (2.6)	0	22 (84.6)	4 (15.4)	0.656
My shoulder hurts during my usual daily activities	0	35 (97.2)	1 (2.8)	2 (7.7)	18 (69.2)	6 (23.1)	0.796
My shoulder hurts when I lean on my elbow or hand	1 (2.6)	35 (92.1)	2 (5.3)	0	20 (80)	5 (20)	0.600
My shoulder hurts when I move my arm.	0	38 (100)	0	0	23 (92)	2(8)	**-**
My shoulder hurts when I write or type.	0	38 (100)	0	0	23 (95.8)	1 (4.2)	-
My shoulder is painful when I hold the driving wheel of my car or handle bars of my bike.	5 (13.2)	33 (86.8)		5(20.8)	14 (58.3)	5 (20.8)	0.233
When I lift and carry something my shoulder hurts.	2 (5.4)	29 (78.4)	6 (16.2)	1 (3.8)	13 (50)	12 (46.2)	0.807
During reaching and grasping above shoulder level my shoulder hurts.	2 (5.3)	32 (84.2)	4 (10.5)	0	17 (68)	8 (32)	0.779
My shoulder is painful when I open or close a door.	0	37 (97.4)	1 (2.6)	0	25 (96.2)	1 (3.8)	0.835
My shoulder is painful when I bring my hand to the back of my head	0	34 (89.5)	4 (10.5)	0	19 (73.1)	7 (26.9)	0.540
My shoulder is painful when I bring my hand to my buttock	1 (2.6)	36 (94.7)	1 (2.6)	0	21 (80.8)	5 (19.2)	0.061
My shoulder is painful when I bring my hand to my low back.	2 (5.3)	34 (89.5)	2 (5.3)	0	19 (73.1)	7 (26.9)	0.503
I rub my painful shoulder more than once during the day.	1 (2.6)	36 (94.7)	1 (2.6)	1 (4)	17 (68)	7 (28)	0.563
Because of my shoulder pain I am more irritable and bad tempered with people than usual.	0	37 (97.4)	1 (2.6)	1 (3.8)	17 (65.4)	8 (30.8)	0.331

* marginal homogeneity test. Pain is unchanged in almost all questions of the SDQ, and more present when lying on the shoulder.

Table S5. Shoulder pain examined with a shoulder disability questionnaire (SDQ) in patients with continuous levobupivacaine analgesia before and one year after surgery.

	**Before surgery N (%)**			**One-year follow-up N (%)**			*P**
	NA	No	Yes	NA	No	Yes	
I wake up at night because of shoulder pain.	0	33 (84.6)	6 (15.4)	1 (4.2)	18 (75)	5 (20.8)	0.291
My shoulder hurts when I lie on it.	1 (2.6)	33 (84.6)	5 (12.8)	0	18 (78.3)	5 (21.7)	0.839
Because of pain in my shoulder, it is difficult to put on a coat or a sweater	1 (2.6)	37 (94.9)	1 (2.6)	0	22 (91.7)	2 (8.3)	0.901
My shoulder hurts during my usual daily activities	0	33 (84.6)	6 (15.4)	0	20 (83.3)	4 (16.7)	0.453
My shoulder hurts when I lean on my elbow or hand	0	35 (89.7)	4 (10.3)	0	21 (87.5)	3 (12.5)	0.648
My shoulder hurts when I move my arm.	0	36 (92.3)	3 (7.7)	0	24 (100)	0	-
My shoulder hurts when I write or type.	0	38 (97.4)	1 (2.6)	0	22 (91.7)	2 (8.3)	-
My shoulder is painful when I hold the driving wheel of my car or handle bars of my bike.	1 (2.6)	36 (92.3)	2 (5.1)	6 (25)	15 (62.5)	3 (12.5)	0.109
When I lift and carry something my shoulder hurts.	0	31 (79.5)	8 (20.5)	2 (8.3)	17 (70.8)	5 (20.8)	0.153
During reaching and grasping above shoulder level my shoulder hurts.	0	32 (82.1)	7 (17.9)	0	20 (87)	3 (13)	0.260
My shoulder is painful when I open or close a door.	0	37 (94.9)	2 (5.1)	0	24 (100)	0	-
My shoulder is painful when I bring my hand to the back of my head	0	32 (82.1)	7 (17.9)	1 (4.3)	21 (91.3)	1 (4.3)	0.629
My shoulder is painful when I bring my hand to my buttock	0	35 (89.7)	4 (10.3)	1 (4.2)	22 (91.7)	1 (4.2)	0.752
My shoulder is painful when I bring my hand to my low back.	0	34 (87.2)	5 (12.8)	1 (4.2)	23 (95.8)	0	-
I rub my painful shoulder more than once during the day.	0	35 (89.7)	4 (10.3)	0	21 (87.5)	3 (12.5)	0.567
Because of my shoulder pain I am more irritable and bad tempered with people than usual.	0	35 (89.7)	4 (10.3)	0	22 (95.7)	1 (4.3)	0.746

* marginal homogeneity test. No statistically significant differences were found between the groups.

Table S6 – Hand grip strength before surgery, 4 days, and one year after surgery.

**Hand grip strength on operated side**	**Diclofenac (N=41)**	***P*†**	**Levobupivacaine bolus (N=39)**	***P*†**	**Levobupivacaine PCA (N=40)**	***P*†**	***P*‡**
before surgery	0.38 (0.34 – 0.44)		0.37 (0.31 – 0.44)		0.40 (0.34 – 0.47)		0.452
4 days after	0.37 (0.30 – 0.43)	0.125	0.35 (0.27 – 0.46)	0.263	0.38 (0.30 – 0.41)	**0.022**	0.778
1 year after	0.32 (0.25 – 0.43)		0.34 (0.30 – 0.40)		0.39 (0.32 – 0.51)		0.161
**Hand grip strength – non-operated side**							
before surgery	0.37 (0.32 – 0.46)		0.38 (0.30 – 0.43)		0.39 (0.33 – 0.46)		0.686
4 days after	0.37 (0.32 – 0.48)	0.269	0.40 (0.32 – 0.44)	0.734	0.40 (0.32 – 0.45)	0.145	0.888
1 year after	0.35 (0.27 – 0.44)		0.38 (0.29 – 0.42)		0.40 (0.32 – 0.49)		0.457

Median (interquartile range); † Friedman's test; ‡ Kruskal Wallis test

Table S7. Survival of patients operated on for breast cancer after 10 years related to body mass index, ASA status, and type of postoperative analgesia.

	Number (%) of deceased	Number (%) of survivors	Total	Survival	The arithmetic mean of months (95% CI)	Logrank test (*P*)
The survival rate after 10 years	18 (15)	102 (85)	120	85 %	110 (104 – 115)	-
The survival rate after 10 years						
BMI (< 20)	1 (25)	3 (75)	4	75 %	120 (120 – 120)	
BMI (20 – 35)	15 (14)	91 (86)	106	86 %	110 (105 – 116)	0.72
BMI (> 35)	2 (22)	7 (78)	9	78 %	103 (79 – 126)	
The survival rate after 10 years						
ASA 1	1 (10)	9 (90)	10	90 %	120 (120 – 120)	
ASA 2	12 (13)	77 (87)	89	86 %	110 (104 – 115)	0.45
ASA 3	5 (24)	16 (76)	21	76 %	107 (92 – 122)	
The survival rate after 10 years						
Diclofenac	6 (15)	35 (85)	41	85 %	107 (96 – 117)	
Levobupivacaine bolus	3 (8)	36 (92)	39	92 %	117 (113 – 121)	0.36
Levobupivacaine PCA	9 (23)	31 (77)	40	77 %	107 (97 – 117)	
The survival rate after 10 years						
Diclofenac	6 (15)	35 (85)	41	85 %	106 (96 – 117)	0.99
Any levobupivacaine group	12 (15)	67 (85)	79	85 %	112 (107 – 117)	

BMI body mass index, ASA American Society of Anesthesiologists.

Table S8 A survival rate of patients operated on for breast cancer until analysis related to body mass index, ASA status and type of postoperative analgesia.

	Number (%) of deceased	Number (%) of survivors	Total	Survival	The arithmetic mean of months (95% CI)	Logrank test (*P*)
Overall survival until analysis	23 (19)	97 (81)	120	80 %	159 (150 – 168)	
Overall survival until analysis &						0.94
BMI (< 20)	1 (25)	3 (75)	4	75 %	156 (136 – 176)	
BMI (20 – 35)	20 (19)	86 (81)	106	81 %	159 (150 – 168)	
BMI (> 35)	2 (22)	7 (78)	9	78 %	140 (105 – 175)	
Overall survival until analysis &						0.43
ASA 1	1 (10)	9 (90)	10	90 %	163 (154 – 172)	
ASA 2	16 (18)	73 (82)	89	82 %	159 (149 – 169)	
ASA 3	6 (29)	15 (71)	21	67 %	144 (122 – 167)	
Overall survival until analysis &						0.22
Diclofenac	9 (22)	32 (78)	41	78 %	154 (137 – 171)	
Levobupivacaine bolus	4 (10)	35 (90)	39	85 %	162 (154 – 170)	
Levobupivacaine PCA	10 (25)	30 (75)	40	75 %	141 (126 – 157)	
Overall survival until analysis &						0.52
Diclofenac	9 (22)	32 (78)	41	78 %	154 (137 – 171)	
Any levobupivacaine group	14 (18)	65 (82)	79	81 %	151 (142 – 161)	

BMI body mass index, ASA American Society of Anesthesiologists.
